# Multifunctional magnetic soft composites: a review

**DOI:** 10.1088/2399-7532/abcb0c

**Published:** 2020-12-08

**Authors:** Shuai Wu, Wenqi Hu, Qiji Ze, Metin Sitti, Ruike Zhao

**Affiliations:** 1 Department of Mechanical and Aerospace Engineering, The Ohio State University, Columbus, OH 43210, United States of America; 2 Physical Intelligence Department, Max Planck Institute for Intelligent Systems, 70569 Stuttgart, Germany

**Keywords:** magnetic soft materials, stimuli-responsive materials, soft robotics, configurable structures

## Abstract

Magnetically responsive soft materials are soft composites where magnetic fillers are embedded into soft polymeric matrices. These active materials have attracted extensive research and industrial interest due to their ability to realize fast and programmable shape changes through remote and untethered control under the application of magnetic fields. They would have many high-impact potential applications in soft robotics/devices, metamaterials, and biomedical devices. With a broad range of functional magnetic fillers, polymeric matrices, and advanced fabrication techniques, the material properties can be programmed for integrated functions, including programmable shape morphing, dynamic shape deformation-based locomotion, object manipulation and assembly, remote heat generation, as well as reconfigurable electronics. In this review, an overview of state-of-the-art developments and future perspectives in the multifunctional magnetically responsive soft materials is presented.

## 1 Introduction

Stimuli-responsive soft materials with both static and dynamic shape programming as well as reconfiguration capabilities have attracted significant interest as they serve as carriers for easy integration of functions, such as actuation and sensing, which offer great potential for applications in soft robotics [1], reconfigurable structures [2], biomedical devices [3, 4], and morphable electronics [5, 6], etc. Compared to conventional rigid materials, the soft and compliant matrix allows for large and dynamic deformations that enable active and passive physical adaptation to external environmental forces, stimuli, and constraints. Soft stimuli-responsive materials usually contain active fillers or microstructures in various types of soft matrices for functional operations under external stimuli such as light [7–9], temperature [10, 11], humidity [12], electric field [13], and magnetic field [14, 15]. The active components can be stimuli-responsive micro/nanoscale particles, fibers, wires, polymer chains, or chemical groups that can induce mechanical and coupled multiphysical responses of the matrix for various physical behaviors and tunable properties [16–19].

Among all the stimuli-responsive soft materials, magnetic soft composites, or magnetically responsive soft materials, allow untethered, fast, and reversible actuation and locomotion with multiple degrees of freedom (DOF) under a remote magnetic field. The superior performance of magnetically responsive soft materials in designing high power density actuators opens new horizons in soft robotics and biomedical fields [20–22]. The integrations of various types of magnetic fillers and soft matrices provide great versatility in creating different magnetically responsive soft materials, which possess the capabilities of precisely programmable shape transformation [23, 24], locomotion [25, 26], magnetic resonance [27], or remote heat generation [28]. With tunable magnitude, direction, and distribution, the applied magnetic fields can accomplish dexterous manipulation of magnetically responsive soft materials to realize multiple functionalities, which dramatically enhances the application potential of the material systems. Additionally, the magnetic fields can penetrate through a wide range of materials such as air, water, or a human body. Therefore, the magnetic soft composites can be manipulated in small and limited spaces, showing unique application potential in the fields of drug delivery and minimally invasive surgery.

Various functionalities have been achieved with different choices of magnetic fillers, polymeric matrices, and fabrication techniques in recent works. In this review, we will first focus on the recent advances in the material design and structure fabrication of various magnetic soft composites. With the understanding of the different types of magnetic soft composites, we will then orient our discussion on the most common functionalities of magnetic soft composites, including shape morphing and tunable properties, dynamic deformation-based navigation, object manipulation and assembly, heat generation and energy output, as well as reconfigurable electronics. We will also review numerous existing applications and tasks realized by these materials. Lastly, we will provide a short discussion on general magnetic field control.

## 2 Material composition

The functionality of a magnetically responsive material system relies on the material properties, including mechanical, electrical, and magnetic properties of the system. To enable various functionalities such as large shape-morphing, programmable deformation, large actuation force, and tunable physical properties, both materials and structures of the system require meticulous design. Magnetic soft materials incorporate different kinds of magnetic fillers with soft material matrices to form functional composites. With a wide range of filler options (ferromagnetic particles, superparamagnetic particles, permanent magnets, etc) and the polymer matrix options (hydrogels, soft elastomers, shape memory polymers (SMPs), etc.), the components of the magnetic composites can be chosen along with suitable fabrication techniques to achieve preferred functionalities.

### 2.1 Functional magnetic fillers

Magnetic fillers are the crucial components of magnetically responsive soft materials that convert the electromagnetic energy into outputs, including mechanical deformation, work, and thermal energy via inductive heating. With a variety of filler types, such as ferromagnetic (including both hard-magnetic and soft-magnetic) particles, superparamagnetic particles, or permanent magnets to choose from, the designed material systems respond differently to the applied magnetic field.

#### 2.1.1 Soft-magnetic fillers

**Figure 1:**
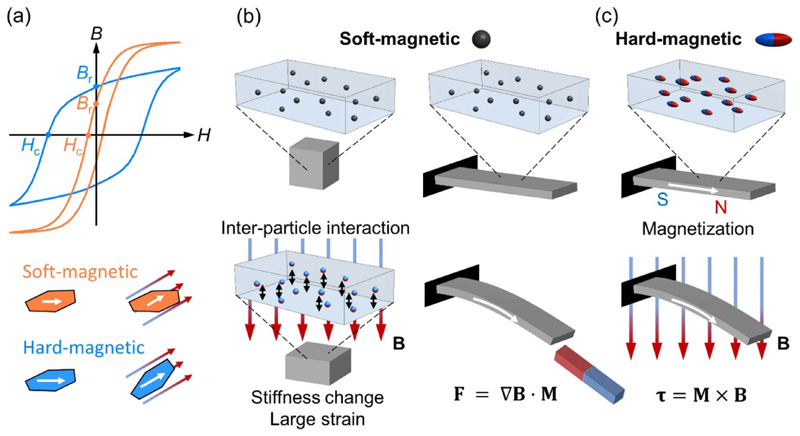
Magnetic particle options commonly used for magnetic soft composites. (a) Magnetic flux density *B* with respect to magnetic field *H* curves of soft-magnetic and hard-magnetic particles. (b) The actuation mechanism of the soft-magnetic composites. (c) The actuation mechanism of the hard-magnetic composites.

In general, soft-magnetic fillers include iron, soft ferrite, iron-silicon alloys, iron-nickel alloys, etc. These materials have relatively high permeability and low coercivity compared to hard-magnetic materials. The magnetic hysteresis loop (magnetic flux density *B* versus applied magnetic field *H*) of soft-magnetic materials is denoted by the orange curve in figure 1(a). The soft-magnetic materials cannot retain strong remanence *B*_r_ after the magnetic field *H* is modified or removed. Due to their relatively low coercivity *H*_c_ (magnetic field required to demagnetize the material), soft-magnetic particles are easily demagnetized and remagnetized to the direction of the applied magnetic field. When applying the magnetic field, the soft-magnetic particles tend to align and show strong interactions due to the high permeability, as shown in figure 1(b). The particle-particle interaction deforms the soft polymeric matrix and leads to the overall deformation of the structure. This behavior is called the magnetostriction. Accompanied by the introduced strain due to the particle-matrix interaction, the stiffness of the structure can be actively tuned by controlling the magnetic field. When applying a gradient field to the magnetic composites, the magnetic body-force per volume generated by the soft-magnetic particles is expressed as

(1) F=∇B⋅M,

in which ∇**B** is the gradient of the applied magnetic flux density, and **M** is the magnetization of the body. The magnetic pulling force can also generate a body torque to bend the beam with locally constrained boundary conditions, such as a fixed end shown in figure 1(b). The controlled strain and stiffness changes of the magnetic composites induced by the magnetostrictive behavior demonstrate various applications such as vibration isolators, absorbers, and sensing devices [29–34]. For example, ferrogels and magnetorheological elastomers (MREs) are commonly fabricated by embedding soft-magnetic particles such as carbonyl iron in hydrogels and elastomers. More detailed discussions about this specific type of magnetic soft composites are also provided in previous reviews [35, 36]. By utilizing the magnetostrictive behavior, these materials behave as actuators and sensors to generate shape changes and detect forces and magnetic fields [37–39]. Over the past decades, works show significant efforts to theoretically describe and predict the magnetostrictive behavior of MREs on the influences of dipole interactions, microstructures, particle distributions, and particle shapes to the macroscopic mechanical behaviors of MREs [40–44]. Mechanics studies on the magnetic soft composites not only help understand the material behavior but also provide a powerful tool for material design.

#### 2.1.2 Hard-magnetic fillers

Lacking the capability to retain magnetization under the external magnetic field, magnetic composites embedded with soft-magnetic particles show relatively low programming flexibility. To achieve good material programmability and realize more complex shape transformations, magnetically responsive materials embedded with hard-magnetic fillers such as neodymium-iron-boron (NdFeB), hard ferrite, alnico alloys, and samarium-cobalt are intensively explored in recent works [24, 45–47]. After being magnetized to saturation (for examples, ~1.6 T for NdFeB and ~0.35 T for hard ferrite) under a relatively large magnetic field, hard-magnetic materials show relatively low permeability compared to soft-magnetic materials, while retaining very high remanence *B*_r_ and large coercivity *H*_c_, as denoted by the blue curve in figure 1(a). When the applied magnetic field (usually much smaller than the field to magnetize the material) is not aligned with the magnetization direction of the hard-magnetic material, magnetic torque is induced to align the material’s magnetization direction with the applied field. When embedding these hard-magnetic particles into soft matrices, the micro-torques will deform the soft composite if there are local constraints such as a fixed end in figure 1(c). Here, the generated torque per volume that leads to elastic deformation of the soft material is expressed as

(2) τ=M×B,

where **B** is the magnetic flux density, and **M**, as previously defined, is the magnetization of the composite. With the programmable magnetization distributions within the hard-magnetic soft composites and the well-controlled external magnetic fields, the resultant torque distribution can facilitate complicated shape transformations [48–50]. In a recent work, efforts have been made by Zhao *et al* on revealing the mechanics of hard-magnetic soft materials [51]. A constitutive model is developed to predict the finite deformation of hard-magnetic soft materials under the application of a magnetic field. The theoretical framework is further implemented in finite element analysis to predict the material’s deformation under magnetic actuation, which also guides the rational designs of both material magnetizations and actuation fields.

Note that when the ferromagnetic particles, including both the hard-magnetic and soft-magnetic particles, are sufficiently small (3–50 nm, depending on materials), superparamagnetism appears [52]. The particles show no remanence *B*r and coercivity *H*c while still possessing the high permeability that helps to provide actuation force as the soft-magnetic particles do under the applied magnetic field. Under a high-frequency alternating magnetic field, the superparamagnetic particles can also be inductively heated and serve as a heat source in the material systems, which is commonly used in the fields of hyperthermia therapy and drug delivery that will be discussed in later sections [53–55].

### 2.2 Functional soft polymeric matrices

The soft robotics and configurable structures generally build upon material systems that are mechanically soft. Different matrices have distinct and specialized properties for various functionalities. Apart from various magnetic fillers, the matrix of the magnetic composites also needs special consideration to achieve targeted functionalities for desired applications. In this section, we will discuss commonly used matrices in four categories: hydrogels, soft elastomers, SMPs, and fluids.

#### 2.2.1 Hydrogels

**Figure 2:**
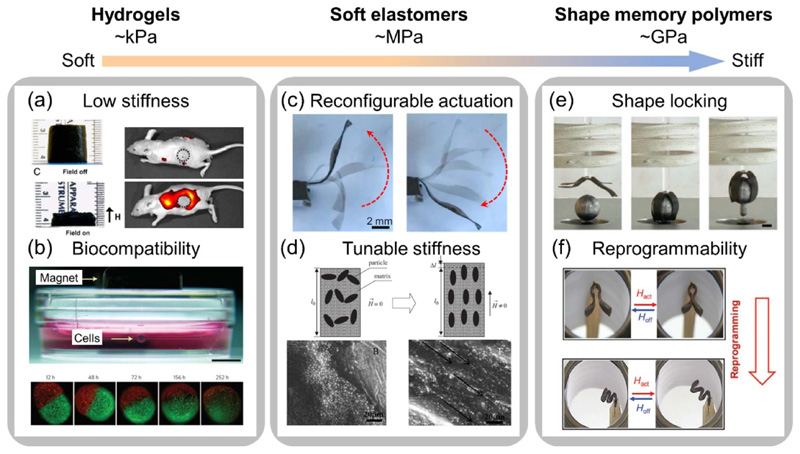
Soft matrix types commonly used for magnetic soft materials with various functionalities. Magnetic hydrogels with (a) low stiffness (Adapted from [61]. Copyright 2011, National Academy of Sciences) and (b) good biocompatibility (Adapted with permission from [64]. Copyright 2010, Springer Nature). Magnetic elastomers with (c) reconfigurable actuation (Reproduced from [48]. Copyright 2016, National Academy of Sciences) and (d) tunable stiffness (Top: Reproduced with permission from [30]. Copyright 2008, Elsevier. Bottom: Reproduced with permission from [67]. Copyright 2018, Elsevier). Magnetic shape memory polymers to achieve (e) shape locking (Adapted with permission from [23]. Copyright 2019, Wiley) and (f) reprogrammability (Adapted with permission from [68]. Copyright 2018, Royal Society of Chemistry).

Hydrogels are a type of polymers that have hydrophilic polymer chains and show elastic behavior. These polymer networks can contain more than 90 wt% of water. Due to their low stiffness, high carrier loading, and good biocompatibility, hydrogels show excellent capabilities in the field of biomedical applications. Incorporation of magnetic particles allows hydrogels to move and deform remotely, which increases their application potential in the fields of soft actuator [47, 56–58], drug delivery [59–63], and cell manipulation [64–66]. The magnetic hydrogels are very soft with stiffness comparable to tissues and organs, which permits large matrix compression or elongation under external magnetic fields. For example, if actuated by a permanent magnet, a porous magnetic hydrogel embedded with Fe_3_O_4_ nanoparticles generates a large deformation with a compressive strain up to 80%, leading to a controlled release of drug and cell agents from the porous scaffold, as demonstrated by the fluorescence images in figure 2(a) [61]. The extraordinary biocompatibility also makes the hydrogels superior in specific biomedical applications. For instance, the magnetic body force induced by Fe_3_O_4_ nanoparticles can levitate cells in the hydrogel under an external magnetic field, realizing a three-dimensional (3D) cell culture with faster cell growing speed and natural protein expression, as shown in figure 2(b) [64].

#### 2.2.2 Soft elastomers

Elastomers are soft polymeric materials with elastic behaviors, which means that they are rubbery materials (Young’s modulus on the order of MPa) whose deformation is reversible under mechanical loads. Due to the softness and high failure strain, soft elastomers are commonly used as the matrix of magnetically responsive materials to allow large deformations. Silicone rubbers such as poly(dimethylsiloxane) and Ecoflex are commercially available examples that do not require complex chemical synthesis. Previous works demonstrate various deformation modes, including elongation, compression, twisting, and bending when embedding hard-magnetic or soft-magnetic particles in the elastomers. With the flexibility to program magnetization, hard-magnetic elastomers permit complex shape morphing capabilities [15, 45, 46, 48, 49, 69]. For example, programmable magnetization enables an artificial cilium with biomimetic motions of complicated recovery and power strokes shown in figure 2(c) [48]. As for MREs, the strong particle-particle interaction and particle-matrix interaction lead to strain and modulus shift, as shown in figure 2(d) [30, 67]. The controlled stiffness changes and the field-dependent properties of MREs are widely used for designing active vibration absorbers and isolators [31–34].

#### 2.2.3 Shape memory polymers

Although the aforementioned soft matrices enable easy shape change under magnetic actuation, they fail to lock the deformed shape without a continuous application of the magnetic field. For applications that need to hold the deformed configurations, this requires continuous power consumption. In these cases, SMPs provide solutions to lock the deformed shape. SMPs are a kind of smart materials that are capable of memorizing temporary shapes and recovering their original shapes under external stimuli such as temperature, light, etc [70–73]. In a thermally triggered SMP, when the temperature is above the SMP’s glass transition temperature *T*_g_, the material is at its rubbery state and is easy to deform. After the material is cooled down below *T*_g_, it turns to the glassy state and stays in the deformed shape. To recover, the SMP is heated to a temperature above the *T*_g_ and returns to its original shape. During the thermally-activated transition of the SMPs, the Young’s modulus generally shifts orders of magnitude from several MPa to several GPa. Many studies have demonstrated approaches to trigger the phase transition and shape memory effect of SMPs by induction heating of magnetically responsive particles in SMP matrices under high frequency alternating magnetic field [28, 68, 74–78]. Carbon nanotubes and Fe_3_O_4_ nanoparticles are commonly used fillers for induction heating and thermal activation of SMPs. Recent studies further integrate the magneto-thermal-responsive behaviors into the so-called magnetic SMPs, where two types of magnetic fields provide driving forces for both deformation and induction heating to change or lock the shape of the material in a controlled manner, as shown in figure 2(e) [23]. Furthermore, complex tasks can be achieved by integrating advanced capabilities such as self-healing and reprogramming into the SMP materials. For example, a reprogrammable SMP combines two kinds of units for actuation and geometry-stabilization, respectively. Applying alternating magnetic fields with different magnitudes can selectively activate the units with different transition temperatures, achieving shape configuration or shape reprogramming shown in figure 2(f) [68].

#### 2.2.4 Magnetorheological fluids and particle-filled fluids

Magnetorheological fluid (MRF) usually consists of magnetic particles in fluids such as silicone oil, fluorocarbon, and water. MRFs show magnetostriction behavior or interesting shape changes under the influence of magnetic fields [79]. Unlike the constrained particles in the polymer network, the distributed particles can move freely in the fluid to instantaneously form chain-like structures under the applied magnetic field, showing a strong magnetorheological effect. Some works develop MRF-filled 3D-printed structures to actively tune the stiffness and damping coefficient of the systems [80–82]. Recent work demonstrates a magnetically responsive composite surface by infiltrating ferrofluid in a porous matrix with microchannels, in which case the magnetic nanoparticles exhibit controllable movement [83]. Under the applied gradient magnetic field, ferrofluid droplets can dynamically morph into multifunctional shapes and configurations, behaving as liquid robots with both cooperative and independent control [84–86].

## 3 Fabrication methods

Both material and geometry designs of the system decide the functionalities of magnetic soft composites. Advanced fabrication methods enable more complex structures and application possibilities. While the molding process shows the capability to fabricate 2D or relatively simple 3D geometries, additive manufacturing techniques, also known as 3D printing [87–89], help achieve rapid fabrications of complex structures of magnetically responsive soft materials. In this section, general fabrication methods of magnetically responsive soft materials will be discussed. A summary of the pros and cons of various fabrication methods is presented in table 1.

### 3.1 Molding

**Figure 3:**
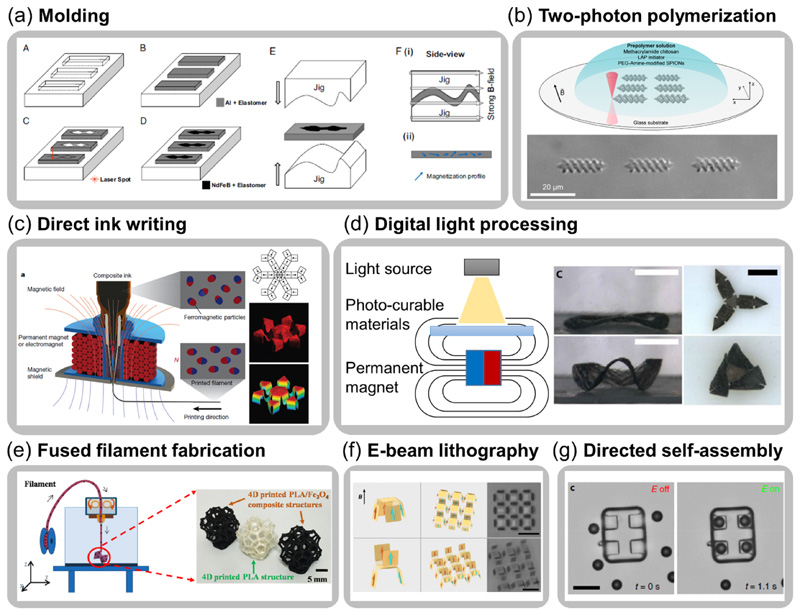
Various manufacturing methods for magnetically responsive soft materials. (a) Molding of silicone rubber embedded with hard-magnetic particles with programmable magnetization (Reproduced from [48]. Copyright 2016, National Academy of Sciences). (b) Two-photon polymerization for the fabrication of small-scale magnetic robots (Adapted with permission from [94]. Copyright 2018, American Chemical Society). (c) Direct ink writing (Adapted with permission from [49]. Copyright 2018, Springer Nature) and (d) digital light processing (Adapted with permission from [95]. Copyright 2019, the Authors, some rights reserved; exclusive licensee American Association for the Advancement of Science) of magnetic soft robots with programmable magnetization. (e) Fused filament fabrication of PLA filament embedded with Fe_3_O_4_ particles (Reproduced with permission from [96]. Copyright 2019, Elsevier). (f) Microfabrication of configurable magnetic structure by e-beam lithography (Adapted with permission from [24]. Copyright 2019, Springer Nature). (g) Field-directed self-assembly of magnetic microactuator by dielectrophoretic forces (Reproduced with permission from [97]. Copyright 2019, Springer Nature).

Molding is a widely used fabrication process of both hard-magnetic composites and soft-magnetic composites that is accomplished by mixing magnetic fillers and polymeric precursors thoroughly and curing to form specific shapes or structures in molds. Anisotropic composites with chain-like microstructures due to the particle alignment can be introduced under the application of magnetic fields during the curing process [90–92]. Materials embedded with hard-magnetic particles can be magnetized after solidification by applying a large magnetic field to form a distributed magnetization [15, 46, 48, 93]. This programming method generally requires a well-designed fixture or fixing strategy to deform the material into the desired actuation shape before being magnetized. For example, as shown in figure 3(a), a beam composed of the inactive part (elastomer with aluminum particles) and active part (elastomer with NdFeB particles) is sandwiched between two fixtures with a predetermined profile [48]. This process helps assign a continuously varied magnetization into the composite beam.

### 3.2 Additive manufacturing

Additive manufacturing, or 3D printing, is a fabrication method used to construct an object layer by layer or filament by filament. Various types of printing methods have been recently developed for the fabrication of magnetically responsive soft materials, realizing not only complex structures from micrometer scales to centimeter scales but also programmable magnetization distribution. Two-photon polymerization has been explored in recent works to enable the microscopic fabrications of magnetic composites [94, 98–100]. Laser pulses are focused into photo-curable materials spatially and temporally to activate material polymerization of microstructures [101]. For example, as shown in figure 3(b), the biocompatible and biodegradable composite precursor polymerizes at the laser focal point, fabricating magnetic hydrogel robots with various dimensions by controlling the laser focus trajectories [94]. This technique allows the fabrication of numerous small and intricate 3D structures with submicron resolution [102–104]. This process greatly enhances the application potential of magnetic composites in biomedical fields.

Direct ink writing (DIW) printing is an extrusion-based additive manufacturing method by which materials with shear-thinning properties are extruded from printing nozzles. It is a flexible filament-by-filament printing method that serves as a tool for fabricating single or multi-material geometries, including magnetic hydrogels [105], elastomers [49, 106], and SMPs [78, 107] with complex structures and programmed magnetization distributions. Recent work develops a customized DIW printing system with *in-situ* magnetization programming capability, where the magnetizations of NdFeB particles are aligned along the extrusion direction under a localized magnetic field. By controlling the printing direction, structures with complicated geometries and magnetization distributions can be fabricated for desired functional deformations under the external magnetic field (figure 3(c) [49]). Digital light processing (DLP) is a printing method where photo-curable materials are exposed to a light pattern and crosslinked layer by layer. For DLP printing of magnetically responsive soft materials, resins with magnetic fillers can be selectively exposed to light source under a controllable magnetic field, programming magnetization during printing [95, 108–111]. The flexibility to print structures with both in-plane and out-of-plane magnetizations helps realize functional actuators shown in figure 3(d) [95].

Other types of 3D printing methods also provide alternative options for the fabrication of magnetically responsive materials. For example, by mixing Fe_3_O_4_ particles into melted poly-lactic acid (PLA), researchers utilize fused filament fabrication to print PLA-based magnetic SMP filaments into 3D structures with remote-activated shape change under alternating magnetic field (figure 3(e) [96]). Recently, a customized inkjet printing system enables the rapid fabrication of multi-material structures with different mechanical, magnetic, and optical properties for designed magnetic actuation [112].

### 3.3 Other fabrication methods

Conventional microfabrication methods possess the ability to manufacture magnetic soft machines with the submicron resolution. For example, e-beam lithography effectively fabricates panels carrying single-domain nanomagnet array connected by hinge springs, as shown in figure 3(f) [24]. Taking advantage of reprogrammable magnetization of the nanomagnets, multiple modules of the 2D structures morph into several 3D patterns under the external magnetic fields. The superiority of the conventional micro/nanofabrication approach grants the proposed approach a resolution down to the nanometer scale. Apart from fabricating a single entity, recent efforts also realize assembled soft machines from given building blocks composed of various functional units and materials, providing an alternative option for creating microscale magnetic soft machines. For example, as shown in figure 3(g), non-magnetic body and magnetic wheels accomplish self-assembly under the guidance of dielectrophoretic forces induced by a gradient electric field [97]. To fabricate even smaller magnetic soft machines, the chemical-aid assembly can be used. In previous work, magnetic particles are connected by DNA to form a chain structure [113]. This structure can undulate under an oscillating magnetic field to function as a microscopic swimmer. In the future, there is still a large space to improve the fabrication of magnetic soft machines at the micrometer and even nanometer scale, attaining arbitrary 3D structures with advanced multifunctionalities.

## 4 Function and operation of magnetic soft materials

**Figure 4:**
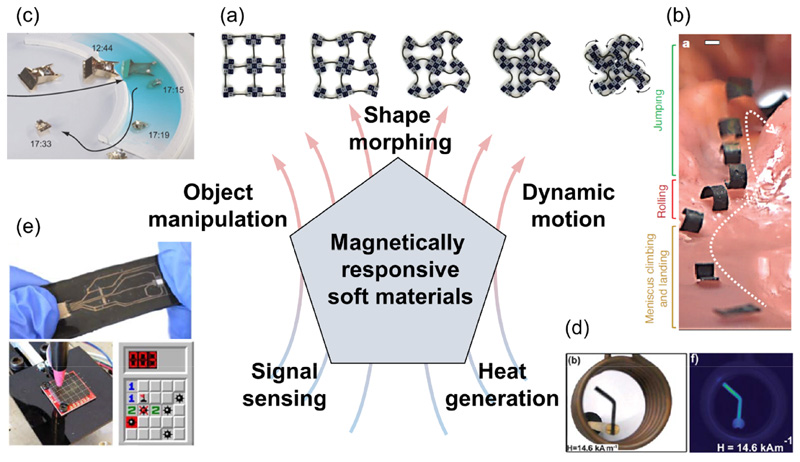
Multifunctionality of magnetically responsive soft materials, including (a) shape morphing (Reproduced with permission from [114]. Copyright 2019, the Authors, some rights reserved; exclusive licensee American Association for the Advancement of Science), (b) dynamic motion (Reproduced with permission from [15]. Copyright 2018, Springer Nature), (c) object manipulation (Reproduced with permission from [115]. Copyright 2017, the Authors, some rights reserved; exclusive licensee American Association for the Advancement of Science), (d) heat generation (Reproduced with permission from [74]. Copyright 2010, Royal Society of Chemistry), and (e) signal sensing (Reproduced with permission from [117]. Copyright 2019, Wiley) capabilities.

By integrating magnetically responsive fillers with various types of polymeric matrices, and by taking advantage of advanced fabrication techniques, excellent design flexibility can be achieved. Under an external magnetic field from permanent magnets or electromagnetic coils, the magnetic fillers can convert input electromagnetic energy into elastic energy, kinetic energy, and/or thermal energy for diverse functionalities. The programmable magnetic properties, including particle alignment and magnetization distribution, allow controllable shape morphing under the applied magnetic field, such as the metamaterial with dexterous shape transformations in figure 4(a) [114]. Navigation is an essential function of magnetically responsive soft materials, where the locomotion of the magnetic composite is implemented through complex dynamic motions such as crawling, rolling, jumping, and climbing that are controlled by tuning the magnitude and direction of the magnetic field (figure 4(b) [15]). With the capability of shape configuration and navigation under magnetic fields, magnetic composites are proven to be ideal candidates for delicate object manipulation [115, 116], as illustrated by the robot in figure 4(c). Under a high frequency alternating magnetic field, certain types of magnetic particles can generate a large amount of heat by inductive heating, and the generated heat can serve as direct energy output in the hyperthermia treatment of tumors. Heat can also be coupled with other physics of magnetic composites for functions such as the remote activation of shape memory effect, as demonstrated in figure 4(d) [74]. Moreover, the coupled behaviors of magnetic materials and magnetic field, as well as the multiphysics couplings of developed magnetically responsive soft materials, provide new strategies for configurable electronics and signal sensing (figure 4(e) [117]).

### 4.1 Programmable shape morphing and tunable properties

**Figure 5:**
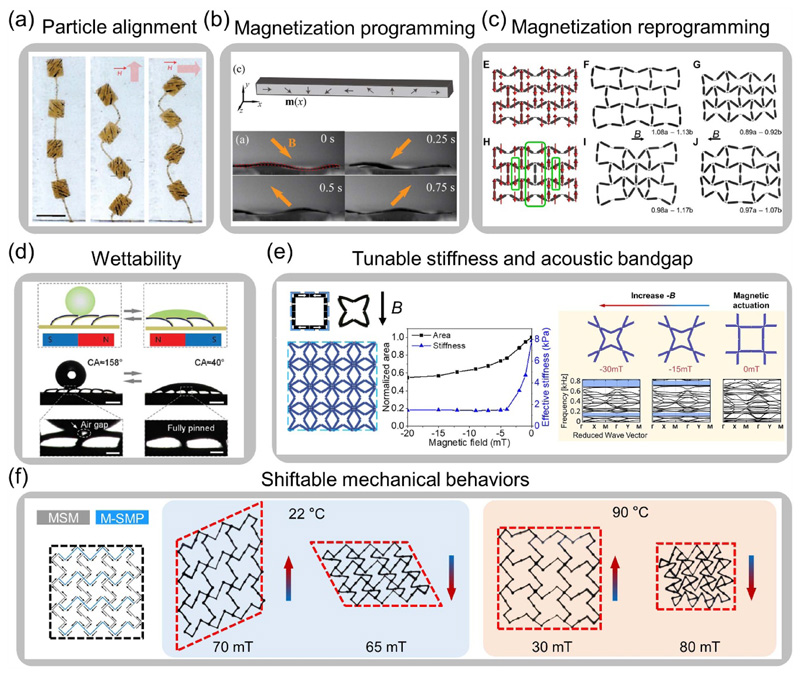
Programmable shape morphingand tunable properties of magnetic soft composites. (a) Actuator with predesigned particle alignment distribution (Reproduced with permission from [108]. Copyright 2011, Springer Nature). (b) Magnetic soft materials programmed with continuous magnetization for undulatory wave configurations (Reproduced with permission from [45]. Copyright 2014, AIP Publishing). (c) Magnetic auxetic metamaterial with encoded discrete magnetization and reprogrammable deformations (Reproduced with permission from [118]. Copyright 2020, the Authors, some rights reserved; exclusive licensee American Association for the Advancement of Science). (d) An array of Janus microplates with controllable wettability (Reproduced with permission from [119]. Copyright 2019, Wiley). (e) Auxetic metamaterials with actively tunable stiffness (Left: Adapted with permission from [46]. Copyright 2019, American Chemical Society) and acoustic bandgaps (Right: Adapted with permission from [120]. Copyright 2020, Wiley). (f) Active metamaterial with shiftable mechanical behaviors under the cooperative thermal and magnetic stimuli (Adapted with permission from [107]. Copyright 2020, American Chemical Society).

Well-designed magnetic soft materials exhibit controllable responses to an applied external magnetic field for large deformation as programmed. One advantage of magnetic soft composites is the programmability of magnetization distribution through magnetic particle alignment. As shown in figure 5(a), the Fe_3_O_4_ nanoparticles are aligned when regionally polymerizing the photo-curable resin, which assigns spatially distributed magnetic anisotropy in the actuator [108]. The alternating particle chain directions help the actuator achieve two wavy shapes by switching magnetic field directions. Another strategy to program the magnetic property is to assign the magnetization distribution of hard-magnetic materials for complex shape morphing. For example, by assigning a non-uniform and continuously changing magnetization distribution, a beam demonstrates a complicated time-varying undulatory deformation under a rotating magnetic field, as shown in figure 5(b) [45]. Exploring reprogrammability of magnetic soft composites also improves the shape morphing capability for reconfigurable deformations. For example, a recent effort demonstrates a magnetization reprogramming strategy by increasing the temperature above the embedded CrO_2_ magnetic microparticles’ Curie temperature and cooling the magnetic soft composites with a reprogramming field on. This strategy enables on-demand magnetization erasing and rewriting with the assistance of localized heating, which augments the configurability and adaptability of active metamaterials, as shown in figure 5(c) [118].

The properties and functionalities of the soft material systems are related to the configurations and deformations of the structures. With the ability to undergo large deformations as programmed, the mechanical [46, 80, 107, 121], optical [112, 122], and acoustic properties [120, 123–127], or wettability [106, 119] of magnetic soft composites are actively tunable under the applied magnetic field. For instance, an array of microplates embedded with aligned Fe microparticles possess a superhydrophobic or hydrophilic surface on each side of the plates (figure 5(d) [119]). The array can bend to one side or the other by changing the direction of the magnetic field, allowing the water droplet to either freely roll or pin on the structure’s surface. As shown in figure 5(e), by designing structure geometries and magnetization distributions, an auxetic metamaterial shows actively controllable stiffness down to 20% of the initial stiffness under the external magnetic field [46]. With a considerable area shrinkage, both the number and the magnitude of the acoustic bandgaps can be tuned by tailoring the magnetic field [120]. Figure 5(f) illustrates an active metamaterial system with the structural integration of magnetic soft materials and magnetic SMPs. Its multiphysics responses under the cooperative thermal and magnetic stimuli enable shiftable mechanical behaviors such as shear, biaxial contraction and expansion, and bending, which considerably enhance the versatility of the metamaterial’s shape-changing capability [107]. An array of dipole elements can modify tilting angles under a relatively small magnetic field, and the configurable structure innovatively demonstrates tunable resonant frequency as an electromagnetic filter [128]. In recent work, an optimization algorithm is utilized to assign magnetic and non-magnetic polymers with distinct transmissions to different locations, realizing desired actuation and optical appearance under the applied magnetic field [112].

### 4.2 Dynamic deformation-based motion and navigation

**Figure 6:**
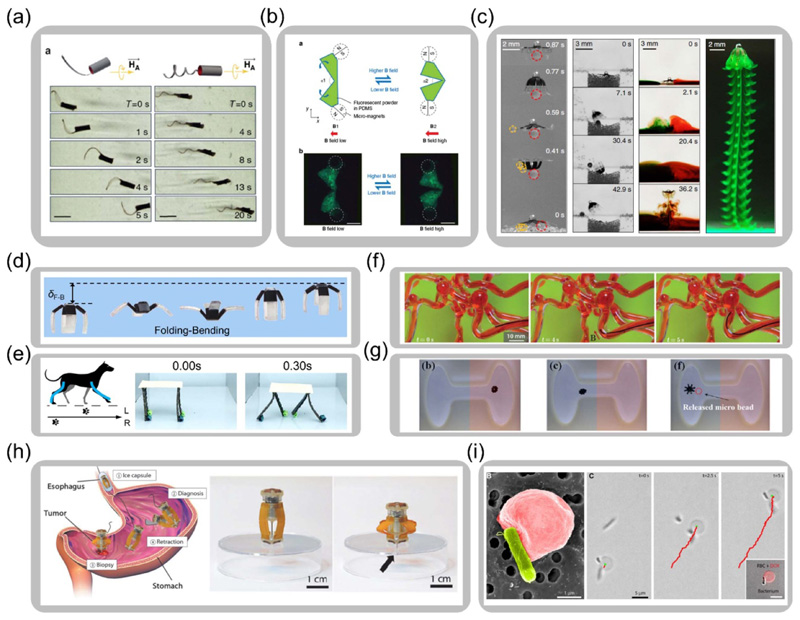
Magnetic soft composites for functional navigation. (a) Bio-inspired swimming robots embedded with aligned magnetic particles for self-folding (Reproduced with permission from [109]. Copyright 2016, Springer Nature). (b) Biomimetic scallop swimming robot (Reproduced with permission from [129]. Copyright 2014, Springer Nature). (c) Multifunctional bio-inspired magnetic jellyfish robot (Reproduced with permission from [69]. Copyright 2019, Springer Nature). (d) Magnetic swimming robot with asymmetric actuation for combined folding-bending deformation (Adapted with permission from [46]. Copyright 2019, American Chemical Society). (e) Biomimetic dog trot gait of a magnetic robot with synergistically controlled legs (Adapted with permission from [130]. Copyright 2020, Wiley). (f) Steering and navigation of a magnetically responsive catheter in a vascular model (Reproduced with permission from [22]. Copyright 2019, the Authors, some rights reserved; exclusive licensee American Association for the Advancement of Science). (g) Magnetic microgripper with controlled navigation and drug release (Reproduced from [63]. Copyright 2016, IOP Publishing). (h) Magnetically controlled capsule robot with the fine-needle biopsy capability (Reproduced with permission from [131]. Copyright 2020, Mary Ann Liebert, Inc.). (i) Bio-hybrid swimming robot guided by the magnetic field (Reproduced with permission from [132]. Copyright 2018, The Authors, some rights reserved; exclusive licensee American Association for the Advancement of Science).

Compared to other stimuli such as light, heat, or pH value, the magnetic field generated by electromagnetic coils can be regulated rapidly and precisely with preferred magnitude, direction, and distribution. With well-designed time-dependent uniform, gradient, oscillating, or rotating magnetic fields, the functional morphing and shape-changing capabilities of magnetic soft composites are further enhanced to realize dynamic motion and navigation under real-time control. For example, as shown in figure 6(a), magnetic-driven hydrogel microswimmers inspired by bacterial movements can attain controllable mobility under a rotating magnetic field [109]. By designing embedded magnetic particle alignments, different microrobot morphologies can be programmed with varied swimming performances. Figure 6(b) illustrates a microswimmer embedded with two magnets to assign discrete magnetization [129]. By applying a periodical magnetic field, the microswimmer reorients the magnets around a hinge to achieve reciprocal motions, mimicking scallop swimming. Apart from programming particle alignment and discrete magnetization, assigning continuous magnetization distribution enhances the versatility of dynamic deformation-based motion. For example, a bio-inspired jellyfish robot is designed with complex structures and magnetization distribution. The miniature swimming robot actively controls surrounding flows under different magnetic fields, accomplishing various tasks such as carrying cargos, searching for objects, and mixing chemicals, as shown in figure 6(c) [69]. Another work introduces asymmetric actuation in a swimming motion by combining folding and bending deformations for energy-efficient propelling, as illustrated in figure 6(d) [46]. Such highly efficient dynamic motions rely on cooperative dynamic deformations of different functional components in a robot. Recently, an evolutionary algorithm-based design strategy is developed to guide the functional design and fabrication of the soft magnetic actuators. The shape changes of legs of a trotting dog are taken as the target shapes for magnetic actuations, which determine magnetization distributions for the magnetic actuators to achieve the biomimetic two-beat walking motion, as shown in figure 6(e) [130].

With a wide range of magnetic fields from centimeter scales to human-sized scales, the controllable motion and navigation of magnetically responsive materials are highly applicable for biomedical purposes such as cell culture [64, 133–135], drug deliveries [60–62, 136–138], and minimally invasive surgeries [20, 139–143]. The untethered and fast control also makes dexterous operations of magnetic soft composites possible in a small and confined space such as the brain, stomach, liver, or vascular system. For instance, a small-scale hard-magnetic composite fiber coated with a layer of biocompatible hydrogel skin is recently presented. The robot steers and navigates within a complicated and confined vascular model by controlling the applied magnetic field magnitude and direction, as displayed in figure 6(f) [22]. The controlled navigation capability of magnetically responsive soft materials lays the foundation for complex operations and multi-tasking abilities. A bi-layer magnetic hydrogel gripper, which navigates under a gradient magnetic field, is able to switch between folding and unfolding states under different pH values to release drugs on demand, as illustrated in figure 6(g) [63]. A microneedle that is fabricated by rolling up a magnetic polymer sheet can achieve controlled walking motion and drilling task on biological tissues [143]. With an attached permanent magnet, an origami-based robot achieves operations such as unfolding, locomotion, and releasing drugs to the wound area in the stomach model under the application of magnetic field [144]. Previous work demonstrates a soft capsule robot with magnetically controlled navigation and fine-needle biopsy capabilities. It successfully shows improvements of the diagnosis accuracy of the tumor by taking deep tissue samples under the surface of the gastrointestinal tract, as shown in figure 6(h) [131]. To realize even higher adaptability, recent works break the boundary between natural lives and artificial machines. Bio-hybrid systems of engineered cells with designed properties are demonstrated to navigate and release drugs controlled by the magnetic field [132, 145–149]. For example, bio-hybrid microswimmers are constructed from red blood cells and bacteria with improved biocompatibility, load-carrying ability, and configurability. The bacterium propels the robot through the magnetic pulling force from iron oxide nanoparticles that are loaded in the red blood cell, as demonstrated in figure 6(i) [132].

### 4.3 Object manipulation and assembly

**Figure 7:**
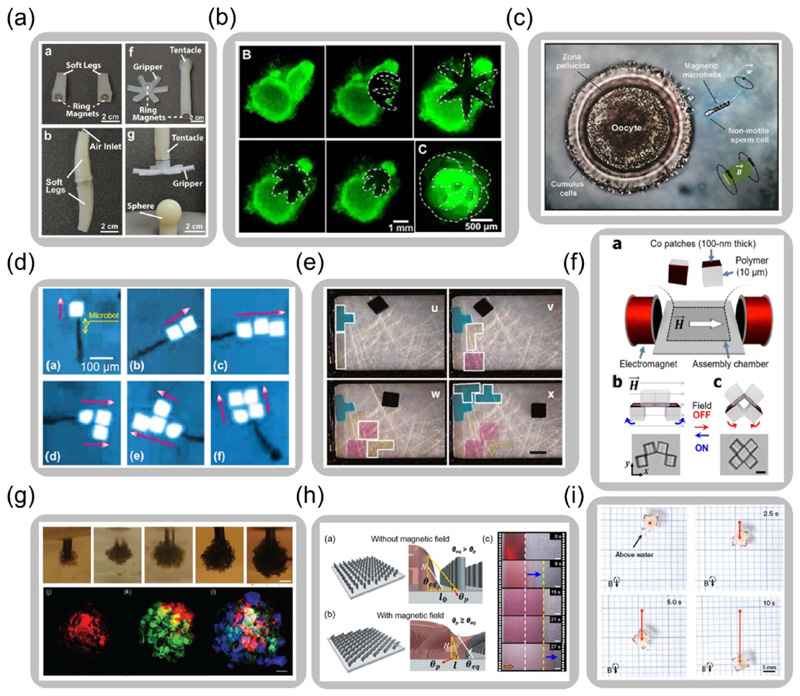
Manipulation of magnetic soft composites for functional assembly. (a) Part assembly of soft pneumatic robots by magnetic dipole interaction (Reproduced with permission from [150]. Copyright 2013, Wiley). (b) Magnetically and thermally controlled soft gripper grasping and cutting a cell cluster (Adapted with permission from [66]. Copyright 2015, American Chemical Society). (c) Capturing, transporting, and releasing of an immotile sperm cell via a magnetic robot (Reproduced with permission from [151]. Copyright 2016, American Chemical Society). (d) Different 2D structures assembled by the same building module via a magnetic robot (Reproduced with permission from [152]. Copyright 2010, Wiley). (e) Assembly of 3D structures with different shapes of building modules via a magnetic robot (Reproduced with permission from [153]. Copyright 2014, Springer Nature). (f) Magnetic field-assisted self-assembly and biomimetic swimming motions (Reproduced with permission from [154]. Copyright 2020, American Chemical Society). (g) Three-dimensional assembly of magnetic hydrogels embedded with cells (Adapted with permission from [65]. Copyright 2011, Wiley). (h) Cooperative behaviors of magnetic pillars to spray liquid (Reproduced with permission from [155]. Copyright 2020, American Chemical Society). (i) A swarm of robots to move an object cooperatively under the application of magnetic field (Adapted with permission from [156]. Copyright 2019, Springer Nature).

Modular assembly, widely used in modern industry and product design, is an approach to use independent and functional subdivisions or modules to integrate into customizable systems. Modularity provides design flexibility while requiring different groups of modules with well-designed functionalities and reliable inter-module connections for assembly. For magnetically responsive materials, the interactions between the magnetized materials, such as the dipole-dipole interaction of magnetic materials, provide a reliable assembly strategy. For example, as shown in figure 7(a), pneumatic actuators with different geometries and functions assemble into different robot assemblies by interactions between embedded magnets, allowing rapid robot prototype design or damaged part replacement [150].

The ability for the magnetically responsive materials to collect, operate, and assemble objects, makes the magnetic soft composites powerful tools for object manipulation and modular assembly due to the well-controlled transformation and transportation. For example, under the guidance of the magnetic field and controlled manipulation of thermal and pH conditions, a millimeter gripper goes through large and reversible deformations, showing the ability to grasp and excise a cluster of cells, as displayed in figure 7(b) [66]. At an even smaller scale, a magnetically driven robot succeeds in capturing, transporting, and releasing of an immotile sperm into a targeted egg, as shown in figure 7(c) [151]. Figure 7(d) illustrates a magnetic robot with properly regulated transportation and object delivery capability, which helps build different microstructures from metallic plate modules [152]. Furthermore, a method is demonstrated to precisely program complicated 3D geometries with adjustable morphologies and chemical features composed of different building modules via the modular assembly by a magnetic microrobot, as illustrated in figure 7(e) [153].

Apart from being utilized as the object manipulators, the magnetic soft composites themselves can serve as building modules due to their versatile and programmable multiphysical properties. Under a well-controlled magnetic field, magnetic modules can achieve functional self-assembly for preferred architectures. For instance, as shown in figure 7(f), microcubes assemble into a chain structure as magnetic coatings on the cubes are aligned under the applied magnetic field [154, 157]. After assembly, each half of the four-cube structure rotates up and down around the center connection under a periodic magnetic field, achieving a biomimetic reciprocal swimming motion [154]. Figure 7(g) illustrates a bottom-up assembly strategy that collects different magnetic hydrogel modules and forms a multilayer structure, which is embedded with living cells [65]. This building method provides a new possibility to fabricate 3D biological structures for *in-vitro* tissue engineering.

On the basis of controlling one or several objects, the cooperative actions of a group of magnetic modules pave a new avenue to perform certain tasks [155, 156, 158–163]. For example, a method to design, program, and control an array of magnetic pillars simultaneously and systematically is presented. The collective behaviors of the magnetic pillars with synergistic deformations help manipulate and spray the liquid under magnetic control, as shown in figure 7(h) [155]. The cooperative actions by a group of robots expand the capability of the single robot and realize escalated functionality. An example of this is the on-demand control of a swarm of magnetic robots under the applied field as they collectively fulfill tasks such as moving an object, as demonstrated in figure 7(i) [156]. To control a group of objects to function as a whole, it is essential to understand the interactions of each object and the dynamics of the system. For instance, previous work thoroughly studies the dynamic self-assembly of magnetic discs on an air-water interface [163]. By modifying the disc morphologies, the pairwise interactions between the discs alter due to capillary force change, leading to different programmable magnetically guided assemblies.

### 4.4 Heat generation and energy output

**Figure 8:**
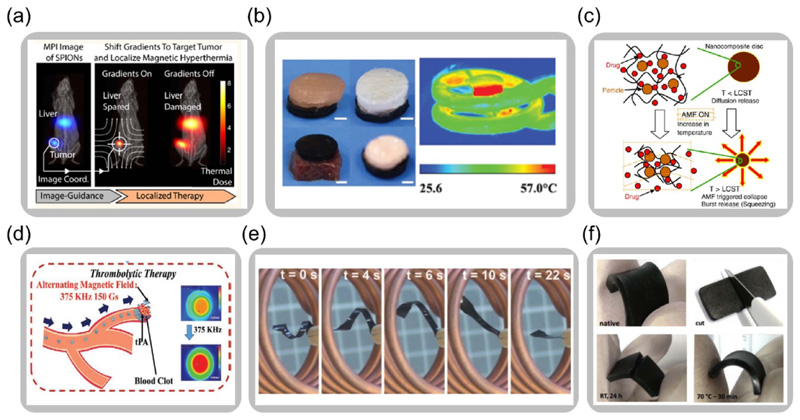
Heat generation and energy output of magnetic soft composites. (a) Localized hyperthermia guided bythe magnetic particle imaging technique (Reproduced with permission from [54]. Copyright 2018, American Chemical Society). (b) Double network magnetic hydrogel for hyperthermia treatment (Adapted with permission from [169]. Copyright 2019, Royal Society of Chemistry). (c) Controlled drug release by inductive heating of magnetically responsive hydrogel (Adapted with permission from [60]. Copyright 2008, Elsevier). (d) Magnetic robot swarm with controlled locomotion and on-demand drug-releasing (Reproduced with permission from [170]. Copyright 2020, Wiley). (e) Magnetic shape memory polymer actuator with remote activation by inductive heating (Reproduced from [28]. Copyright 2006, National Academy of Sciences). (f) Remotely controlled self-healing of magnetic composite with dynamic bonds (Reproduced with permission from [171]. Copyright 2015, Elsevier).

When exposed to a high-frequency alternating magnetic field, magnetic particles dissipate thermal energy due to the magnetic hysteresis loss [164]. The functionality of the magnetically responsive materials to realize effective heat generation can be utilized for various applications. One category of applications is to directly exploit generated heat. Magnetic hyperthermia is an experimental approach to treat cancers by inductive heating of nanoparticles introduced into the tumor [53, 165–168]. Recently, a process is demonstrated to achieve localized hyperthermia treatment under the guidance of magnetic particle imaging. Ferrofluid can be traced and allocated to the targeted region by the applied gradient magnetic field, reducing the collateral damage to healthy organs, as shown in figure 8(a) [54]. Biocompatible composites with magnetic particles embedded in soft matrices are also developed to solve the off-target delivery problem while possessing other specialized properties. For example, a magnetic double network hydrogel exhibits outstanding mechanical properties and magnetic hyperthermia functionality, as shown in figure 8(b) [169].

Apart from directly utilizing the converted thermal energy, inductive heating can help tune the temperature-dependent properties of magnetically responsive soft materials. The generated thermal energy can also couple with multiphysics responses of the various types of soft matrices for shape transformations [172–174], drug deliveries [55], and self-healing [171, 175, 176]. For example, as shown in figure 8(c), under the applied alternating magnetic field, the inductively heated superparamagnetic particles in the magnetic hydrogel lead to matrix temperature increase, releasing drugs due to the matrix shrinkage [60]. The heat generation on an as-needed basis provides more possibilities and flexibilities for the functional operations of the magnetically responsive materials. In recent work, a magnetic microrobot swarm moves to a blood clot using a rotating magnetic field. The loaded tissue plasminogen activator is released under a high-frequency magnetic field, removing the blood clot for noninvasive thrombolysis, as shown in figure 8(d) [170]. Moreover, specialized properties of different polymeric matrices also incorporate inductive heating of magnetic particles for different functionalities. The strategy to actively tune the material’s mechanical properties can provide more flexibility when designing soft robotics and active metamaterials. For instance, a developed magnetic SMP actuator achieves remote activation of shape recovery shown in figure 8(e) [28]. When utilizing dynamic bonds in the polymer network, the self-healing property of the magnetic composite can be remotely activated, as illustrated in figure 8(f) [171].

### 4.5 Magnetic soft composites for electronics

**Figure 9:**
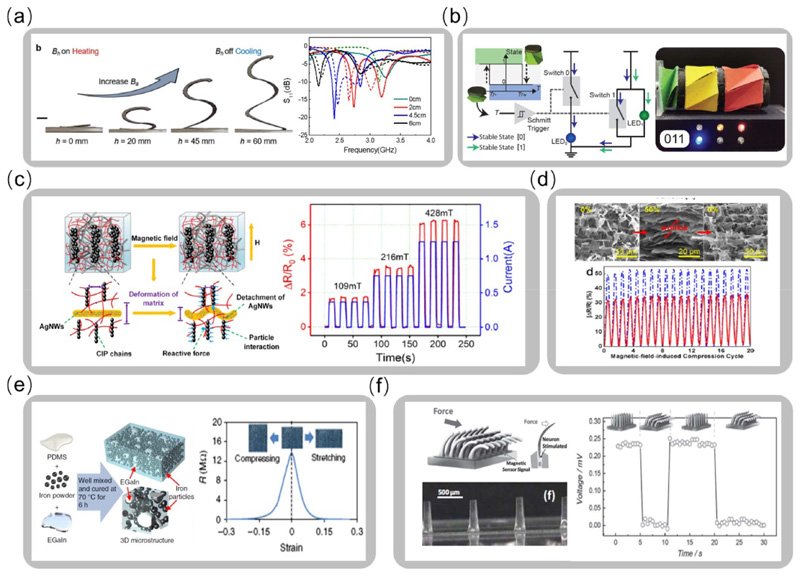
Configurable electronics and sensors via multiphysics coupling of magnetic soft composites. (a) Configurable antenna with an actively tunable resonant frequency (Adapted with permission from [23]. Copyright 2019, Wiley). (b) Multifunctional Magneto-mechano-electric origami assembly for digital computing. (Adapted from [188]. Copyright 2020, National Academy of Sciences). (c) Magnetically responsive soft material sensor by embedding silver nanowires in MRE (Adapted with permission from [189]. Copyright 2018, Elsevier). (d) Self-sensing magnetic graphene aerogel with strain-dependent resistance (Adapted with permission from [182]. Copyright 2015, American Chemical Society). (e) Magnetically responsive soft material sensor embedding liquid metal in MRE (Adapted with permission from [190]. Copyright 2019, Springer Nature). (f) Deformation sensor mimicking cilium structure (Adapted with permission from [191]. Copyright 2015, Wiley).

Due to the ability to configure and modulate properties, flexible electronics show increasing importance in the applications of wearable devices, flexible sensors, and self-sensing soft robotics. When actively morphing the shape under a magnetic field, or passively by mechanical loading, the electromagnetic and electrical properties of magnetically responsive soft materials can be adjusted due to geometry change and particle relocation. Recent works demonstrate the possibility of using magnetically responsive soft materials for flexible electronics such as configurable antennas [177–179], electromagnetic filters [128], electric capacitors [180], inductors [181], and sensors [39, 182–187]. For example, a configurable antenna is made of the recently developed magnetic SMP coated with silver paste, allowing continuous height change with shape locking capability. The resonant frequency of the antenna can be actively tuned by morphing the height under the applied magnetic field, as shown in figure 9(a) [23]. Recent work develops a multifunctional magnetic origami system with coupled magneto-mechano-electric behaviors, as shown in figure 9(b) [188]. By integrating the programmed magnetic soft materials in a homogeneously distributed manner into the bistable origami assembly, the magnetic actuation provides independent control of the folding/unfolding of each unit cell with instantaneous shape locking, which enables various functions such as tunable physical properties and configurable electronics for digital computing.

While the magnetic fillers in the polymeric matrices enable shape transformations and tunable properties, extra functional fillers such as silver nanowires, graphene, and liquid metal can be added to the magnetic composite for enhanced functionalities. For example, by mixing silver nanowire networks into an MRE structure, the magnetic soft material shows coupled mechano-electro-magnetic properties. As shown in figure 9(c), under the external magnetic field, the interaction between magnetic chains leads to deformation and electrical property changes [189]. The resistance of the structure increases to 200% with a magnetic field of 428 mT. The ultralight magnetic aerogels [182] exhibit up to 52% compression strain when applying a magnetic field, and the inner interaction between the graphene walls leads to a maximum of 53% resistance decrease, as shown in figure 9(d). A positive piezoconductive effect is demonstrated by mixing liquid metal droplets into MRE. When applying compression, the resistance of the structure decreases rapidly, as shown in figure 9(e) [190]. Such multiphysics coupling behaviors enable the self-sensing capability of magnetic composites. For example, an artificial cilium structure embedded with iron nanowires in the polymeric columns can detect mechanical deformations by exporting electrical signal changes, as shown in figure 9(f) [191]. With various mechano-electro-magnetic properties to design and exploit, magnetic composites exhibit wide application potentials and pave the way to achieve the self-sensing, integrated decision-making, and closed-loop control of intelligent and self-adaptive magnetic soft material systems.

## 5 Magnetic control

The programmable and controllable behaviors of the magneic soft composites rely on the external application of magnetic fields. A permanent magnet is the most straightforward and easy-handling source of the magnetic field with tailorable direction and magnitude by adjusting the relative orientation and distance to the object manually or via a robot arm. Most of the applications require predictable and precisely controlled actuation and motion of the magnetic composite, which need reliable and precise programming of the robotic arm to move the magnets [192–194]. Various electromagnetic coils or array magnets are also commonly implemented [15, 23, 95, 195–198]. These strategies provide magnetic fields with controllable direction, magnitude, gradient, and frequency for functional operations of the magnetic composites free of mechanical moving systems.

### 5.1 Uniform magnetic fields

Uniform magnetic fields are usually applied to achieve predictable deformations of the magnetic composites without generating a magnetic pulling force [109, 129]. A Halbach array, which assembles permanent magnets in a ring pattern, produces a static uniform magnetic field within the ring [198, 199]. Helmholtz coils are widely adopted to generate uniform magnetic fields by a pair of circular coils, in which the applied current determines the direction and magnitude of the magnetic field [15, 23, 95]. Sophisticated motions can be realized by changing the external magnetic field in time. Superposition of the Helmholtz coils in three orthogonal axes creates the 3D magnetic fields which enable static or dynamic shape deformations in 3D.

### 5.2 Non-uniform magnetic fields

In addition to a uniform **B** field, a field that is spatially non-uniform can generate more DOFs. For example, we can assume that three **B** field components (*B*_x_, *B*_y_, *B*_z_) are not uniform while their five independent first-order derivatives, i.e. the gradients terms (*B*_xx_, *B*_xy_, *B*_xz_, *B*_yy_, *B*_yz_), are uniform [48, 195, 200]. Note that *B*_zz_ is not an independent term as it satisfies *B*_xx_+*B*_yy_+*B*_zz_ = 0. With such an assumption, these five gradient terms can be additionally included to control the magnetic soft composites. In the work of Lum *et al* [48], all eight independent **B** field parameters (*B*_x_, *B*_y_, *B*_z_, *B*_xx_, *B*_xy_, *B*_xz_, *B*_yy_, *B*_yz_) and the magnetization profile of the magnetic soft materials (*M*_x_, *M*_y_, *M*_z_) are given as the design inputs, and an automatic computational optimization approach is formulated to generate a set of desired values for these design inputs, which can realize targeted kinematics for a fix-free beam structure made of magnetic soft composite. In order to control these eight **B** field parameters independently, at least eight electromagnetic coils must be used. Previous work has reported an optimized configuration of these eight coils towards achieving sufficient magnetic gradient force in all spatial directions [195]. By using a similar formulation and different optimization cost function, coil configurations for other applications can also be achieved.

To achieve even more complex spatial variation in the **B** field, the assumption on the uniform gradient terms can be further relaxed, and higher-order derivatives of the **B** field can be introduced. This could potentially be very useful in creating complex control signals. For example, it can enable different magnetic gradient forces on a piece of magnetic soft material even with a homogeneous magnetization profile and consequently induce various deformations. However, such introduction of higher-order derivatives also means more electromagnets or permanent magnets should be used to control these newly introduced DOFs. Along this line, an example of pioneering efforts has been done by Dong *et al*, in which 2D arrays of permanent magnets are used to program different magnetic potential maps [158]. One pre-programmed permanent magnet array could simultaneously control many magnetic agents constrained on either the liquid or solid surfaces. To change the magnetic potential map, another pre-programmed 2D array must replace the existing one. Such a constraint could be addressed in the future as the permanent magnets used could be swapped by smaller and more powerful electromagnets.

Due to the natural availability of the gradient coil and its imaging capacity, the magnetic resonance imaging (MRI) system has attracted significant attention in controlling untethered magnetic devices for medical applications [201]. The gradient coil unit in a typical MRI system consists of two sets of Golay coils and one set of Maxwell coil [202]. This gradient coil unit is designed to scan through the working area to sample the magnetic properties, which are used later to reconstruct the 3D image of the sample. Due to its nature as a measurement tool, the gradient coil is designed, fabricated, and calibrated very precisely. Thus, it can generate any accurate controlled magnetic gradient force in 3D space within its maximum strength. However, it should be noted that the main **B** field of MRI is very strong. For example, 3 T has been the mainstream for those MRIs deployed in the hospitals while 1.5 T system does exist, and 7 T system is under development [203]. Such a development towards a higher main **B** field is driven by the fact that a stronger main **B** field leads to a higher spatial resolution of the MR imaging [204]. Such a strong **B** field could be problematic for those magnetic soft composites that function through their magnetization profiles. This is because such a large **B** field may remagnetize the magnetic soft composite. Even if the composite can survive the remagnetization process, it would quickly deform and stay at a specific stable configuration as the torque induced by this main **B** field is huge. Since this main **B** field is also designed to be very constant and not vary temporally, the deformed composite cannot change its shape dynamically and consequently loses its mechanical characteristics as soft material. Therefore, the magnetic soft material should be selected very carefully to make the composite if applications with MRI are desired, or it will simply function as a rigid body without desired deformation. To improve the impact of the magnetic soft composite in MRI application, new materials with a higher coercivity field, or novel actuation mechanism for magnetic soft composites should be developed in the future.

## 6 Conclusion and perspectives

**Figure 10:**
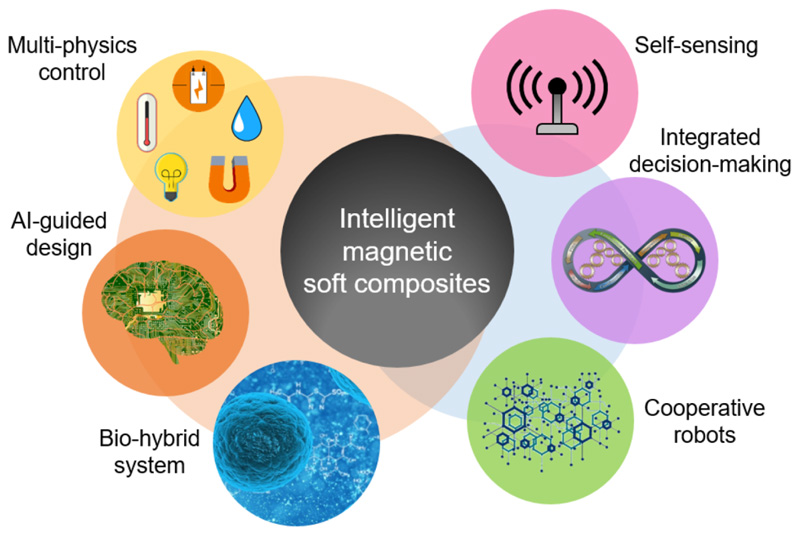
Future perspectives to make magnetic soft composites intelligent and autonomous.

In this review, we focus on state-of-the-art works on magnetically responsive soft materials and the multifunctionality of the material systems, including shape morphing with tunable properties, dynamic motion and navigation, object manipulation and assembly, heat generation and energy output, as well as soft and configurable electronics. Numerous recent advances of magnetic soft composites in the fields of soft robotics, biomedical devices, active metamaterials, and electronics are discussed. Although possessing the merits of remote-controlled capability, fast actuation speed, and flexible programmability, developed magnetic soft composites still possess limitations. For example, the magnetic soft composites are more suitable for microscale to centimeter-scale applications at the current stage. The magnetic soft composites with even larger dimensions may lose their advantages due to the actuating systems’ bulkiness. The external magnetic field also affects all magnetically responsive materials within the field simultaneously. It requires extra efforts to achieve both on-demand localized and global controls by specially designed material properties, more complex magnetic actuations, and multi-physics control methods. Thus, more efforts should take place to promote soft magnetic composites for real applications in an intelligent way. There are two main trends for the coming studies to work on to elevate the system complexity and capability to the next level, as illustrated in figure 10.

From the material perspective, magnetically responsive soft materials still have a large space for further exploration even though possessing all the advantages discussed above. Combining the advantages of different material systems by multiphysics control with the response to light, temperature, electric field, humidity, and magnetic field, one may obtain enhanced soft materials with multifunctionality that adapt to different application scenarios. Also, the flexibility to program the material property as well as the applied magnetic field, poses certain application difficulties of magnetically responsive soft materials. One emerging field utilizes optimization algorithms and artificial intelligence (AI) to guide the design and control of the material systems. Taking the magnetic soft robots as an example, to satisfy the high complexity level of the desired robotic motion, the material system requires rationally designed physical (mechanical, magnetic, etc) properties and external control, which might be difficult to implement through the manual approaches, or the so-called brute-force approaches. As a tool to enlarge the application potentials of magnetically responsive soft materials, AI-guided design strategy helps to make the materials even more intelligent to use. With the development of techniques, the boundary between machines and lives becomes vague. A simple cell can be viewed as an intricate machine that provides countless inspirations and extraordinary functions. In recent works, bio-hybrid systems that combine living cells, tissues, or animals with artificial materials provide new possibilities for designing and controlling multifunctional devices, paving the way for more complex and adaptive soft robots.

From the system perspective, autonomous control of the stimuli-responsive materials, which enables materials themselves to sense, react, and achieve programmed tasks on their own, makes the systems intelligent; however, most of the current works are still at the stage of open-loop control. The abilities to integrate self-sensing, decision making, and closed-loop control, make the systems perform complicated tasks autonomously. By taking advantage of emerging hardware and algorithms, intelligent magnetically responsive material systems can become a reality. Moreover, with the operation of a single piece of magnetically responsive soft materials being well understood, the capability to manipulate multiple magnetic robots closely and cooperatively is essential in future works. This requires good communication between multiple objects and reliable coordination to function as a whole system. With the development of the system, centralized control of different objects synergistically and collaboratively leads to intelligent magnetically responsive soft materials for enhanced functionalities.

## Figures and Tables

**Table 1:** State-of-the-art fabrication methods for magnetic soft composites.

Methods	Materials	Pros	Cons
Molding	Thermal-curable/Photo-curable resins	Simple fabrication; Programmable magnetization; Scalability	2D and relatively simple 3D geometries
Two-photon polymerization	Photo-curable resins	High resolution (sub-micron)	High cost
Direct ink writing	Thermal-curable/Photo-curable resins	Versatile material types; Multi-material printing; Programmable magnetization during printing	Low resolution; Postprocessing required
Digital light processing	Photo-curable resins	High resolution; Programmable magnetization during printing; Fast fabrication	Single material (generally); Particle sedimentation
Fused filament fabrication	Thermoplastics	Multi-material printing	Low resolution
Inkjet printing	Photo-curable resins	Multi-material printing; Fast fabrication	Complex control
Microfabrication	—	High resolution (sub-micron)	High cost
Micro-assembly	—	Programmable assembly of functional units	Complex control
